# The Adaptive Evolution in the Fall Armyworm *Spodoptera frugiperda* (Lepidoptera: Noctuidae) Revealed by the Diversity of Larval Gut Bacteria

**DOI:** 10.3390/genes14020321

**Published:** 2023-01-26

**Authors:** Yan-Ping Wang, Xu Liu, Chun-Yan Yi, Xing-Yu Chen, Chang-Hua Liu, Cui-Cui Zhang, Qing-Dong Chen, Song Chen, Hong-Ling Liu, De-Qiang Pu

**Affiliations:** 1 Key Laboratory of Integrated Pest Management of Southwest Crops, Institute of Plant Protection, Sichuan Academy of Agricultural Sciences, Chengdu 610066, China; 2 Service Center of Sichuan Academy of Agricultural Sciences, Chengdu 610066, China

**Keywords:** *Spodoptera frugiperda*, coevolution, 16S rDNA, gut bacteria diversity, bacterial functional analysis

## Abstract

Insect gut microbes have important roles in host feeding, digestion, immunity, development, and coevolution with pests. The fall armyworm, *Spodoptera frugiperda* (Smith, 1797), is a major migratory agricultural pest worldwide. The effects of host plant on the pest’s gut bacteria remain to be investigated to better understand their coevolution. In this study, differences in the gut bacterial communities were examined for the fifth and sixth instar larvae of *S. frugiperda* fed on leaves of different host plants (corn, sorghum, highland barley, and citrus). The 16S rDNA full-length amplification and sequencing method was used to determine the abundance and diversity of gut bacteria in larval intestines. The highest richness and diversity of gut bacteria were in corn-fed fifth instar larvae, whereas in sixth instar larvae, the richness and diversity were higher when larvae were fed by other crops. Firmicutes and Proteobacteria were dominant phyla in gut bacterial communities of fifth and sixth instar larvae. According to the LDA Effect Size (LEfSe) analysis, the host plants had important effects on the structure of gut bacterial communities in *S. frugiperda*. In the PICRUSt2 analysis, most predicted functional categories were associated with metabolism. Thus, the host plant species attacked by *S. frugiperda* larvae can affect their gut bacterial communities, and such changes are likely important in the adaptive evolution of *S. frugiperda* to host plants.

## 1. Introduction

The fall armyworm, *Spodoptera frugiperda* (Smith, 1797) [1] (Lepidoptera: Noctuidae), is a major migratory agricultural pest of global concern. It originated in tropical and subtropical regions of America, where it is widely distributed [2,3,4]. *S. frugiperda* invaded Ghana and Nigeria in southwest Africa in 2016 [5,6] and was later reported in India in 2018 [7]. The pest was subsequently recorded in Sri Lanka, Thailand, Myanmar, and other Asian countries [8]. The armyworm invaded Yunnan in southern China in 2018 and then rapidly expanded to most areas of the country [9,10,11]. *S. frugiperda* is an omnivorous pest with strong migratory ability, a high rate of reproduction, and a short life cycle. It damages 353 species of plants in 76 families, including the major crops corn, sorghum, sugarcane, barley, rice, pepper, wild oat, and potato [12]. Maize crops can suffer serious economic loss when attacked [13]. However, information on the effective prevention and control strategies for *S. frugiperda* is lacking. *S. frugiperda* has developed resistance to a variety of insecticides, including diamide and neonicotinoids [14]. Because *S. frugiperda* is a newly invaded major agricultural pest in China, native natural enemy insects are being investigated to provide biological control [15]. Nevertheless, these control measures might be harmful to the environment and not economically viable. Therefore, there is a great need for alternative green control methods for *S. frugiperda*.

Insect gut microbiomes are important for the digestion of food, absorption of nutrients, and general metabolism [16,17]. The gut and associated microbes resist invasion and colonization by external pathogens [18,19], degrade harmful substances, and produce drug resistance [20]. Communities of gut bacteria can promote host absorption and utilization of food [21], and different foods can also affect the composition and metabolic function of gut bacterial communities [22]. Although many plant tissues are low in nutrients, indigestible, or toxic, herbivorous insects are among the most numerous and diverse groups of organisms [23,24]. Herbivorous insects have numerous morphological, behavioral, and physiological characteristics that enable them to overcome dietary barriers [25,26,27]. Some insects can adapt to new host plants, and in that process, changes occur in the abundance and composition of gut enzymes that reduce the toxicity of plant allelochemicals [28,29,30]. Therefore, the composition and diversity of gut bacterial communities have been one of the recent hotspots of entomological research. Enriching the understanding of the coevolution between insects and their gut bacteria can provide a theoretical basis for pest control [31,32,33].

The host plant is an important factor affecting insect gut bacteria. The feeding habits of insects impact the composition and structure as well as the diversity and function of gut bacterial communities [34]. Gut bacterial communities have been investigated in an increasing number of insects, including bees [35,36], fruit flies [37,38], beetles [39], termites [40], and other common pests [41,42,43,44]. In ground beetles, their food habits and habitats affect their gut bacterial and fungal communities [39]. In cluster analysis of relative abundances of orthologous gene clusters, high similarities were observed among wood- and litter-feeding termites, but those groups had strong differences with humivorous species [40]. In a cockroach pest, the gut bacteria are highly dynamic, and bacterial communities reassemble relatively rapidly and with different compositions in a diet-specific manner (the highest diversity was associated with a no-protein diet) [41]. The flexibility of the gut bacteria is most likely due to the fact that cockroaches are omnivorous with variable diets [41]. In a comparative analysis of the moth pests’ midgut bacterial diversity, the plant species influenced the composition of the gut bacterial community; the moth larvae reared on an artificial diet and different host plants revealed significantly different compositions and diversity of gut bacterial communities [42]. Thus, host plants can greatly influence the composition and structure of gut bacterial communities in pests, which may be essential in long-term adaptation to host plants [43]. Similarly, gut bacterial communities of another moth pest are influenced by the host diet and therefore may also be important in adaptation to the hosts [44].

Although the gut bacteria of *S. frugiperda* have been examined previously [45,46,47,48], how feeding on different host plants affects the composition and functions of gut bacterial communities is not fully clear. In this study, *S. frugiperda* larvae were reared on leaves of corn, sorghum, highland barley, and citrus. Then, 16S rDNA sequence amplification was used to compare the effects of different host plants on the structure, diversity, and functions of gut bacterial communities in *S. frugiperda*. The results will provide a foundation to generate new ideas for further study of the effects of host plants on gut bacterial communities of *S. frugiperda* and the adaptive evolution of this important pest. In addition, new insights may lead to the manipulation of gut bacterial communities for pest control of *S. frugiperda*.

## 2. Materials and Methods

### 2.1. Insect Collection and Laboratory Feeding

The samples of *S. frugiperda* were collected from a corn field in the Base of Xindu, Sichuan Academy of Agricultural Sciences, Chengdu, China. The larvae were fed on an artificial diet containing the following contents (g/L): soybean powder (225), wheat powder (125), yeast (40), casein (20), cholesterol (0.6), and agar (30) [49]. Insects were reared at our laboratory for three generations at 27 ± 1 °C with 70 ± 5% relative humidity, and a light:dark = 16:8 h photoperiod. Larvae were separately reared on leaves of corn, sorghum, highland barley, and citrus in the laboratory. Host plants included corn (*Zea mays* L. var. Chengdan 11, ZmL), sorghum (*Sorghum bicolor* L. Moench. Chuannuo 15, SbL), highland barley (*Hordeum vulgare* L. var. Kangqing 9, HvL), and citrus (*Citrus reticulata* Blanco. Chunjian, CrB). Plants were cultivated to the 3–4-true-leaf stages. Newly hatched larvae were reared to 5th and 6th instars on fresh young leaves of the four different host plants, respectively. All experiments and procedures for this study complied with the current animal ethics guidelines and did not involve any protected animals. 

A total of 32 gut samples of 5th and 6th instar larvae of *S. frugiperda* fed on different host plants were collected and profiled. Thus, the experiment had two treatment factors: host plant and larval stage. Host plants were corn (Zm), sorghum (Sb), highland barley (Hv), and citrus (Cr), and larval stages were 5th instar (L1 or B1) and 6th instar (L2 or B2). Therefore, there were eight treatment combinations: ZmL1, ZmL2, SbL1, SbL2, HvL1, HvL2, CrB1, CrB2.

### 2.2. Processing of S. frugiperda Larvae

To ensure the gut bacteria were in a relatively stable state, the *S. frugiperda* larvae were transferred to new centrifuge tubes and starved for 24 h in a natural environment. After all materials were prepared, dissections were performed on an ultra-clean bench. First, beakers were prepared with sterile water and absolute ethanol. Larvae were removed from centrifuge tubes, soaked in absolute ethanol for 90 s, and then blotted on filter paper. Larvae were then washed three times with sterile water, blotted dry, and placed in petri dishes. Under a stereomicroscope, the head of a larva was held with pointed tweezers, and medical scissors were used to cut along the abdomen below the mouth. Ganglion, salivary glands, martensitic ducts, fat bodies, and other organs were carefully removed. Then, the intestine was completely removed, placed in a sterile centrifuge tube, quickly frozen with liquid nitrogen, and stored at −80 °C.

### 2.3. DNA Extraction and 16S rDNA Sequencing

To extract the total DNA from gut contents, a PowerSoil DNA Isolation Kit (MOBIO Laboratories Inc., Carlsbad, CA, USA) was used according to the protocol provided by the manufacturer. The integrity of extracted DNA was confirmed by agarose gel electrophoresis. Extracted DNA was quantified in a Qubit 2.0 (Invitrogen, Carlsbad, CA, USA), and 10 ng/uL was used for amplification and sequencing of the 16S rDNA genes from 32 samples. PCR full-length amplification was performed using the 16S primers F (5′AGAGTTTGATCCTGGCTCAG3′) and R (5′GNTACCTTGTTACGACTT3′) with a Phusion^®^ High-Fidelity PCR Master Mix (New England Biolabs Inc., Ipswich, MA, USA) under the following conditions: 94 °C for 5 min; 35 cycles of 94 °C for 30 s, 56 °C for 30 s, 72 °C for 30 s; 72 °C for 5 min. This was followed by product purification, construction of a SMART bel library, and sequencing on PacBio [50]. Total DNA was sent to Beijing Novogene Bioinformatics Technology Co., Ltd. (Beijing, China) for sequencing.

### 2.4. Statistical Analyses

PacBio offline data were exported to a bam file through PacBio’s SMRT analysis software (version 7.0). After samples were distinguished according to barcodes, operational taxonomic unit (OTU) clustering and classification analysis were performed. Sequences that were less than 1340 bp or greater than 1640 bp were removed. Uparse software (http://drive5.com/uparse, accessed on 15 January 2022) was used to cluster the clean reads. The sequences were clustered into OTUs (operational taxonomic units) with 97% identity. Species annotation analysis was performed using the Mothur method with the SSUrRNA database of SILVA (http://www.arb-silva.de, accessed on 15 January 2022). We used MUSCLE (http://www.drive5.com/muscle, accessed on 15 January 2022) to perform the rapid multiple sequence alignment and then obtained all the OTUs’ representative sequences. The subsequent analysis of α diversity and β diversity was based on the standardized data. 

The abundances of OTUs were analyzed according to the results of OTU clustering, and a petal map was prepared. α diversity reflects the abundance and species diversity of sample species. QIIME software v1.9.1 was used to calculate α diversity indices, including Chao1, Simpson, and Shannon. The raw data were tested for normality and homogeneity of variance using the Shapiro–Wilk and Levene’s test, respectively. After log-transforming the data, normality was confirmed (*p* > 0.05) and the data were suitable for parametric analysis. ANOVA (one-way analysis of variance) followed by Tukey’s tests were performed to test the difference between host plants, where the diversity of gut bacteria was the response variable. Differences were considered significant when *p* < 0.05. R software v2.15.3 was used to analyze the differences between groups in the β diversity index, and the LDA Effect Size (LEfSe) analysis was used to test the significance of differences in the composition and structure of bacterial communities in samples from different treatments. Last, PICRUSt2 (https://github.com/picrust/picrust2, accessed on 15 January 2022) was used to predict the metabolic functions of bacterial communities based on the KEGG database (https://www.kegg.jp, accessed on 15 January 2022).

## 3. Results

### 3.1. Sequence Analysis

**Figure 1:**
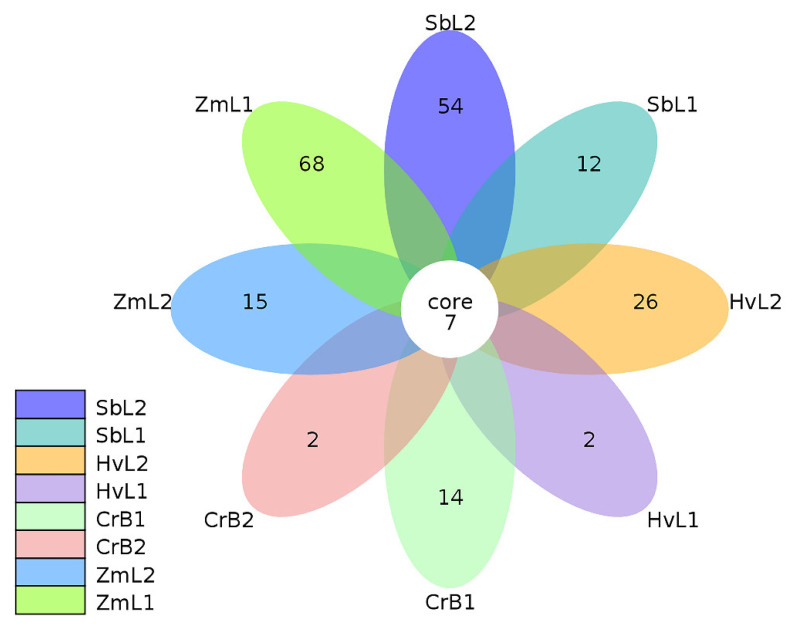
Flower plot of bacterial operational taxonomic units (OTUs) in guts of *Spodoptera frugiperda* larvae fed on leaves of different host plants. Each petal in the flower represents a treatment, and the core number in the overlapped parts of the petals represents the number of OTUs shared among treatments. The numbers at petal edges represent the number of unique OTUs in a treatment. Treatments: letters represent different host plants (Zm, corn; Sb, sorghum; Hv, highland barley; Cr, citrus), and numbers represent different larval instars (L1 or B1, fifth instar; L2 or B2, sixth instar).

Thirty-two *S. frugiperda* gut samples were examined. A total of 568,300 original reads and 748,360,295 bp of original bases were obtained (Table 1). After filtration, 15,802 high-quality average reads and 566 unique average reads were obtained (Figure S1). From the fifth and sixth instar larvae raised on different host plants, 498 and 562 OTUs, respectively, were obtained from sequencing data (Tables S1 and S2). Gut bacteria were classified into nine phyla, 14 classes, 32 orders, 56 families, 93 genera, and 66 species. Differences in the OTUs of gut bacteria in different larval instars fed on different host plants were compared in a flower plot (Figure 1). Although only seven OTUs of gut bacteria were shared among different *S. frugiperda* instars fed on different host plants, they indicated there were similarities in the composition of bacterial communities. In fifth instar larvae of *S. frugiperda*, the number of unique OTUs was 68 in those fed on corn (ZmL1), 14 in those fed on citrus (CrB1), 12 in those fed on sorghum (SbL1), and 2 in those fed on highland barley (HvL1). In sixth instar larvae, the number of unique OTUs was 54 in those fed on sorghum (SbL2), 26 in those fed on highland barley (HvL2), 15 in those fed on corn (ZmL2), and 2 in those fed on citrus (CrB2). Thus, the composition of gut bacterial communities was different in *S. frugiperda* fed on different host plants.

### 3.2. Taxa Annotation and Relative Abundance

**Figure 2:**
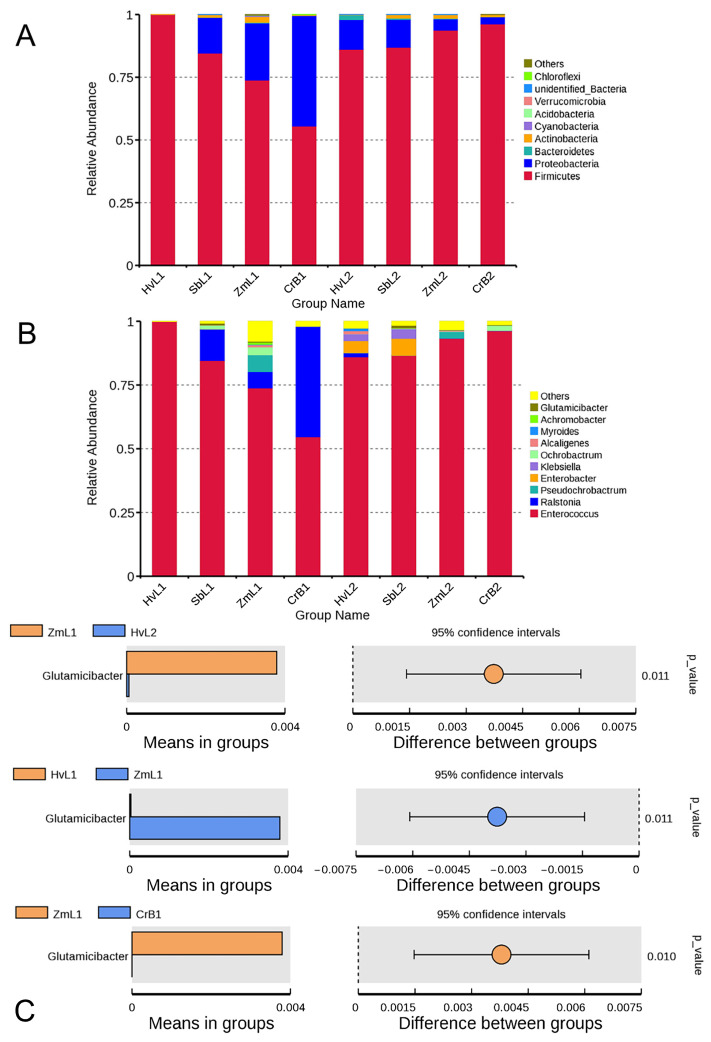
Relative abundance of the most predominant taxa of gut bacteria in *S. frugiperda* larvae fed on leaves of different host plants at the (**A**) phyla and (**B**) genera levels. (**C**) *T*-test analysis of species differences between groups. Others represents the sum of the relative abundances of all phyla (genera) other than the phyla (genera) in the figure. Each bar in the figure represents the mean value of species with significant differences in abundance between groups. It is the *p*-value for the between-group significance test for the corresponding differing species.

The relative abundance of gut bacteria was determined at different taxonomic levels. All samples typically included nine main phyla (Figure 2A and Table S3). In the fifth instar *S. frugiperda*, Firmicutes (avg. 78.48%) was the most abundant phylum among gut bacteria, followed by Proteobacteria (avg. 20.27%), with other phyla at much lower relative abundance, including Bacteroidetes, Cyanobacteria, Actinobacteria, Verrucomicrobia, Chloroflexi, and unidentified Bacteria. In sixth instar larvae, Firmicutes (avg. 90.76%) was also the most abundant phylum, followed by Proteobacteria (avg. 7.57%). Phyla of bacteria were highly consistent between the two instars, with Firmicutes and Proteobacteria dominant in both instars. There were no significant effects of host plants on the phyla of gut bacteria (*p* > 0.05). Although the same phyla were dominant in the guts of the two larval instars, their relative abundances were different.

The dominant genera in fifth instar *S. frugiperda* were primarily *Enterococcus* (avg. 78.26%) and *Ralstonia* (avg. 15.54%), with other genera at a lower relative abundance, including *Pseudochrobactrum*, *Enterobacter*, *Klebsiella*, *Ochrobactrum*, *Alcaligenes*, *Myroides*, *Achromobacter*, and *Glutamicibacter* (Figure 2B and Table S4). In sixth instar larvae, *Enterococcus* (avg. 90.54%) was also the dominant genus, but *Ralstonia* (avg. 0.43%) was less abundant. Whereas *Ralstonia* composed 43.33% of the gut community in the fifth instar larvae fed on citrus (CrB1), the genus composed only 0.03% in the sixth instar larvae fed on corn (ZmL2). The relative abundance of *Glutamicibacter* in the fifth instar larvae fed on corn (ZmL1) was 0.38%, which was significantly different from that on other host treatments, especially in fifth and sixth instar larvae fed on highland barley (HvL1 and HvL2) and fifth instar larvae fed on citrus (CrB1) (*p* < 0.05) (Figure 2C). Thus, the phyla and genera of gut bacteria in *S. frugiperda* reared on different hosts were the same, but relative abundances at each taxonomic level were different.

### 3.3. Diversity of Gut Bacteria

**Figure 3:**
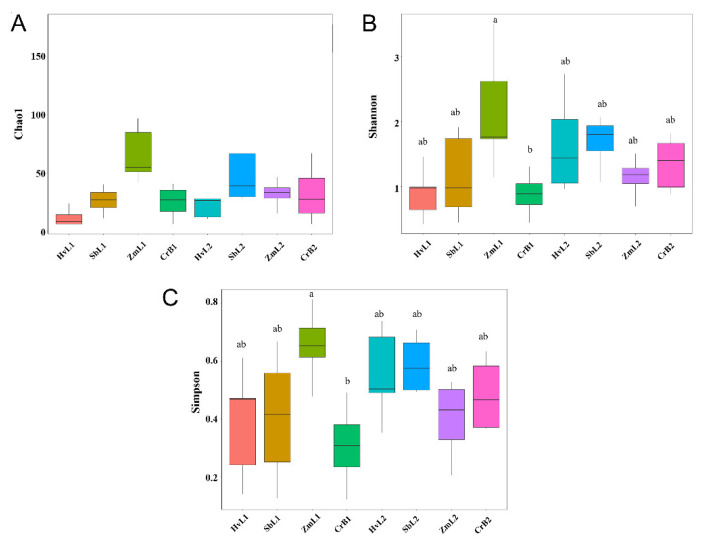
Box plots of (**A**) Chao1, (**B**) Shannon, and (**C**) Simpson α diversity indices of gut bacterial communities in *S. frugiperda* larvae fed on leaves of different host plants. The a and b indicate the significant differences in relative abundance in the same column in the mean values. Different letters above boxes indicate significant differences among treatments (one-way ANOVA, Tukey’s post hoc test) in the mean values.

α diversity of bacterial communities in different treatments was analyzed (Figure 3 and Table S5). The highest Chao index of gut bacteria was 108.34 in ZmL1, followed by 60.073 in SbL2. Thus, the richness of gut bacterial communities was highest in the larvae fed on corn and sorghum. Fifth instar larvae fed on corn (ZmL1) had the highest Shannon and Simpson diversity values (2.153 and 0.654, respectively). Shannon and Simpson indices between ZmL1 and CrB1 were significantly different (*p* < 0.05). Compared with fifth instar larvae fed on corn, the richness and diversity of gut bacteria decreased when larvae fed on leaves of other hosts. Compared with the fifth instar larvae, diversity indices of gut bacterial communities in the sixth instar larvae increased when fed on citrus, sorghum, and highland barley. Thus, there were differences in gut bacterial communities between larval stages in *S. frugiperda*.

**Figure 4:**
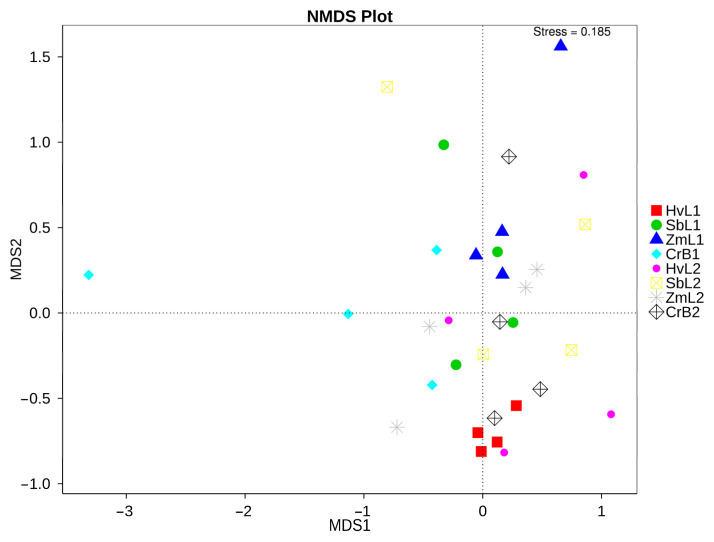
Nonmetric multi-dimensional scaling (NMDS) analysis of gut bacterial communities in *S. frugiperda* larvae fed on leaves of different host plants. Each point in the figure represents a sample; distance between points represents degree of difference, and samples in the same treatment are the same color. When stress is less than 0.2, the NMDS accurately reflects degree of difference between samples.

**Figure 5:**
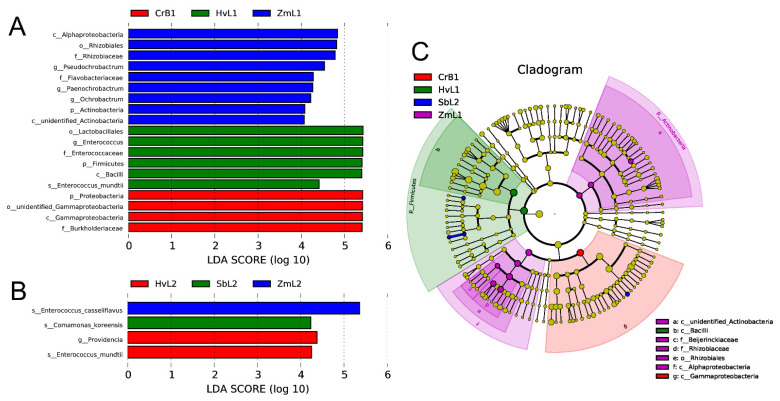
Linear discriminant analysis (LDA) of gut bacterial taxa in *S. frugiperda* larvae fed on leaves of different host plants. Taxa with LDA score (log 10) greater than four in (**A**) fifth and (**B**) sixth instar larvae. (**C**) LEfSe (LDA Effect Size) analysis showing significant differences in bacterial taxa at the level of phylum, class, order, family, and genus, from inside to outside. Small circles at different classification levels represent classifications at a particular level, and their diameters represent relative abundances. Nodes of different colors represent bacteria that were significantly enriched with the corresponding host. Significantly different biomarkers follow the group for coloring. Small yellow nodes indicate bacterial taxa that were not significantly different in guts of larvae fed on different hosts.

To better reflect the nonlinear structure of data on gut bacteria in *S. frugiperda* fed on different hosts, nonmetric multidimensional calibration (NMDS) was performed on sequencing data based on Bray–Curtis distances (Figure 4). The distance between gut bacteria in the fifth instars fed on corn and those in other host plant treatments was relatively large, indicating there were differences in gut bacteria among the different treatments. Differences in gut bacterial communities in fifth and sixth instars fed on different plants were analyzed (Figure 5). In fifth instars fed on corn, the gut bacterial community was significantly enriched from genus to phylum levels. According to linear discriminant analysis effect size (LEfSe), nine bacterial clades were consistently significantly enriched in ZmL1 samples (Figure 5C). Each larval stage had a unique, significantly enriched set of bacteria at taxonomic levels ranging from phylum to species. For example, the genera *Pseudochrobactrum*, *Paenochrobactrum*, and *Ochrobactrum* were notably enriched in ZmL1 when compared with other hosts, whereas *Enterobacter* was notably enriched in HvL1, and *Providencia* was enriched in HvL2. Thus, different bacterial groups were enriched in different larval stages fed on different host plants.

### 3.4. Cluster Analysis of Predominant Bacteria

**Figure 6:**
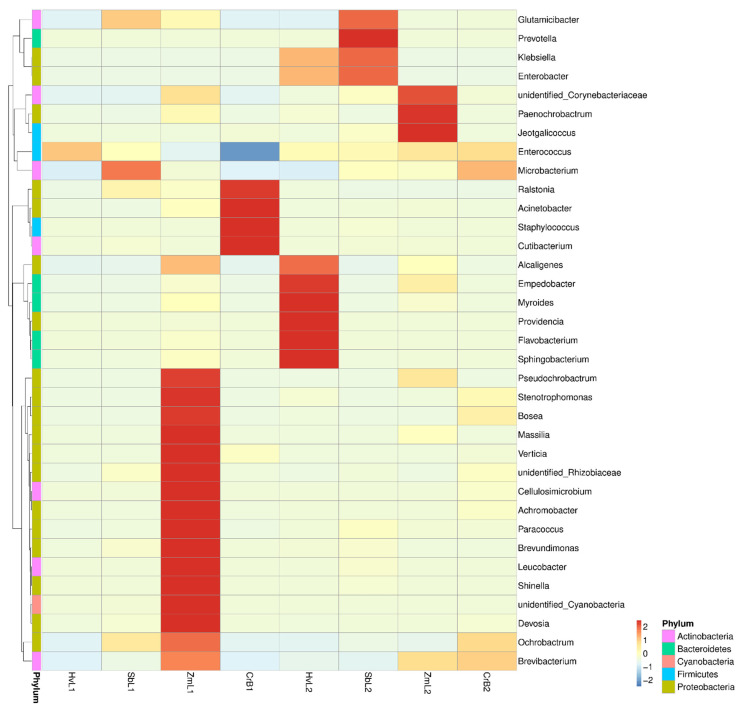
Heat map of relative abundances of the top thirty-five predominant genera of gut bacteria separated by phylum in *S. frugiperda* larvae fed on leaves of different host plants. Treatment names are on the *x*-axis, and genus annotation is on the *y*-axis. The clustering tree for genera is on the left, and heat map values are Z-values obtained after relative abundances of each genus were standardized.

The cluster heat map in Figure 6 shows annotation and abundance information for the top 35 genera based on relative abundance. The genera of gut bacteria in *S. frugiperda* fed on corn (ZmL1, ZmL2), sorghum (SbL1, SbL2), barley (HvL1, HvL2), and citrus (CrB1, CrB2) were clustered in different branches. As shown in the horizontal direction, the abundance of each genus was different in different larval stages fed on different hosts. For both the fifth and sixth instar larvae of *S. frugiperda*, the dominant genera of gut bacteria were also different when reared on different plants.

### 3.5. Prediction of Bacterial Functions

**Figure 7:**
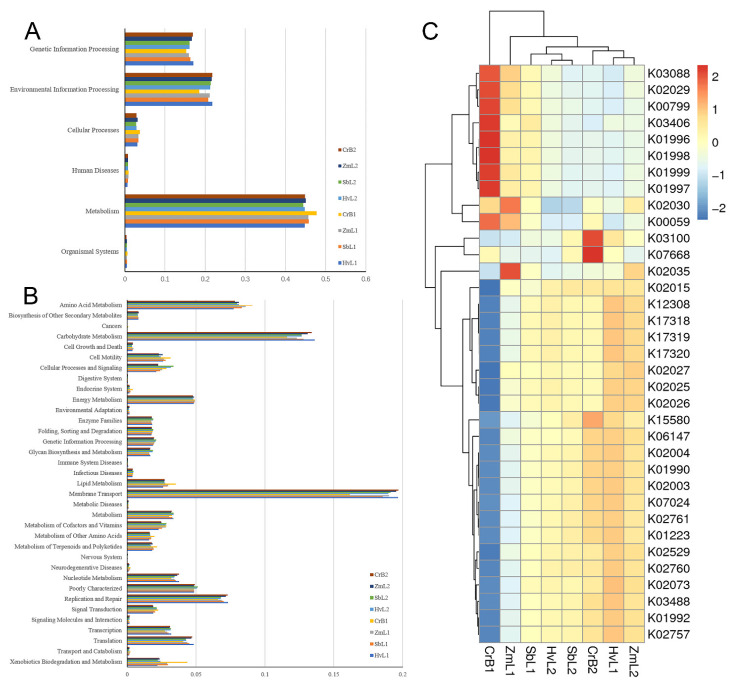
Annotations of KEGG-predicted functions of gut bacterial communities in *S. frugiperda* larvae fed on leaves of different host plants. (**A**) Level 1 and (**B**) level 2. (**C**) PICRUSt2 prediction of proteins based on functions in the KEGG database.

To better understand the important functions of gut bacteria in *S. frugiperda*, relative abundances of Kyoto Encyclopedia of Genes and Genomes (KEGG) pathways were predicted based on the 16S rDNA gene sequences using PICRUSt2. Functions of gut bacteria primarily involved six types of metabolic pathways: metabolism, genetic information processing, environmental information processing, cellular processes, organismal systems, and human diseases (Figure 7A). Gut bacteria primarily functioned in metabolism-associated pathways, which accounted for 45.39 ± 1.07%. In the analysis of the second functional layer of predicted genes (Figure 7B), functions included membrane transport, signal transduction, carbohydrate metabolism, amino acid metabolism, energy metabolism, cell motility, and xenobiotic biodegradation and metabolism among other pathways.

The guts of the fifth and sixth instar larvae of *S. frugiperda* reared on different plants were enriched in different functional proteins (Figure 7C). For example, cold shock protein (K03704) and chitin-binding protein (K03933) were significantly enriched in the fifth instar larvae reared on highland barley (HvL1). Gut microbiomes were enriched in several ABC transporter-related KOs (KEGG Orthogroups), including phosphate and amino acid transporters (K01999), permease protein (K02029, K01997, K01998), ATP-binding protein (K01996), periplasmic binding protein (K01999), hypothetical protein (K02030), and peptide and nickel transporters (K02035). All of the predicted pathways perform the most important functions in the gut and therefore are important in the overall growth and development of *S. frugiperda* larvae.

## 4. Discussion

Multiple external and internal factors could influence the gut microbial community of insect herbivores, such as weather, temperature, usage of antibiotics, host phylogeny, host treatments, diet of insect, host immune recognition, bacterial sources, immigration and competition of microbes, and different developmental stages of insects [51,52,53,54]. Meanwhile, the natural populations of *S. frugiperda* were found to have more diverse gut microbiota and significantly higher diversity of bacteria functional in metabolizing insecticides than laboratory-reared populations [55]. Among these factors, diet or the host plant is the most important factor shaping gut microbiota in *S. frugiperda* [56]. Conversely, the plasticity of the gut microbes can help the insects utilize different foods and enhance the fitness of *S. frugiperda* as a pest.

The omnivorous pest *S. frugiperda* feeds on a wide range of crops, and the analysis of the metagenomic DNA of its gut bacteria can provide the basis for pest control research. However, little is known about how host plants affect the diversity of gut bacteria in *S. frugiperda*. It is essential to identify differences in gut bacteria in *S. frugiperda* feeding on different plants because of their potentially significant effects on larval growth and development. In this study, the total number of OTUs was different in gut bacterial communities of *S. frugiperda* reared on leaves of different hosts, with numbers of OTUs in the larvae fed on corn and sorghum higher than those in larvae fed on highland barley and citrus. This result indicates that different host plants can strongly influence the microbial communities of *S. frugiperda*, similarly to other lepidopterans [57]. Different host plants have variable nutritional content, palatability, and secondary metabolites, which could affect the growth and development of *S. frugiperda* [58]. The significant microbial differences in larvae of *S. frugiperda* fed on different hosts might have resulted from the different nutritional content and inhibitory secondary metabolites in the four hosts used herein. 

In this study, the abundance and diversity of gut bacteria were analyzed in fifth and sixth instar larvae of *S. frugiperda* reared on leaves of corn, sorghum, highland barley, and citrus. We found that the dominant microbes were the same at different larval stages of *S. frugiperda*, but their proportions and compositions were variable. In the α diversity analysis, the abundance and diversity of gut bacteria in HvL1, CrB1, and SbL1 showed an upward trend when compared with ZmL1. There were also differences in the abundance and the diversity of gut bacteria in larvae fed on different plants, and the diversity and abundance of gut bacteria in ZmL1 decreased from the fifth to sixth instars, which may be a result of the presence of one or more inhibitory components in the corn diet provided for the larvae. Such a trend of decrease in α diversity has also been recorded for the larvae of *S. frugiperda* fed on maize leaves in a recent study [52]. Different host plants can cause differences in insect gut microenvironments, which, in turn, lead to differences in gut microbial diversity [59]. The results of this study are consistent with those on other insects. A previous study [60] found differences in dominant flora and their abundances in the guts of a moth pest feeding on three different types of pine trees. Another study [61] found that dietary substrate affects the gut bacteria in cockroaches, with changes in food leading to changes in the dietary matrix available for gut bacteria and, ultimately, changes in gut flora. The inconsistency in α diversity analysis between different studies may be caused by the different sources and treatment of plants, as well as the different sources and rearing conditions of insect samples.

At the phylum level, the composition of gut bacterial communities in fifth and sixth larvae was different among larvae fed on different plants, but Firmicutes was the dominant phylum in both larval stages, followed by Proteobacteria. Previous studies found similar results in *S. frugiperda* [62], and gut bacterial communities of most samples were dominated by Firmicutes. The high abundance of Firmicutes in wild oats is due to better absorption of different nutrients [63]. However, in larvae and adults of *S. frugiperda* from different maize-growing areas in Kenya, Firmicutes was only dominant in one Ngeria (Ngeria-l2) larva and two Kitale (Kitale-m2 and Kitale-m3) adult males [57]. In a closely related lepidopteran pest, *Spodoptera exigua* (Hübner, 1808) [64], the larval gut microbes were found to be 97.9% Proteobacteria and 2.1% Firmicutes [65]. The difference in bacterial proportion between closely related species might have resulted from the different origins of the insect colonies and the remarkable differences in bacterial identification methods [65]. 

At the genus level, *Enterococcus* was the dominant genus of gut bacteria in all eight treatment combinations of *S. frugiperda*. *Enterococcus* is also known as the most common intestinal bacterium in the order Lepidoptera [42,43,44,65]. *Enterococcus* can degrade alkaloids and latex and, therefore, has a stabilizing role in insect tolerance to their toxic diet [63]. In this study, the relative abundance of *Enterococcus* increased significantly with the increase in food intake from the fifth to sixth instars of *S. frugiperda*. The higher abundance of *Enterococcus* could assist digestion and accelerate the development of resistance to insecticides [52]. The dramatic loss of *Ralstonia* from the fifth to sixth instars was also detected in this study. *Ralstonia* has been commonly found in the guts of many insects; it originates from leaf surfaces and comprises important plant pathogens that cause serious damage on a global scale [66,67]. Some studies also speculated that *Ralstonia* may play a vital role in the nitrogen cycle of host insects and the degradation of bactericides [68,69]. However, it is impossible to identify all the *Ralstonia* species accurately, which prevents us from understanding their exact biological function in *S. frugiperda* larvae. The loss of *Ralstonia* from the fifth to sixth instars might be helpful to protect the host plant from being a stable food source for the larvae of *S. frugiperda*. The larval stage of lepidopteran insects suffers the most damage, and changes in larval intestinal microbes can influence the feeding, digestion, growth, and development of these insects [52]. The late larval instars of *S. frugiperda* significantly increase the food intake and their body size grew faster; therefore, the changes in the gut microbiota are associated with the growth and development of *S. frugiperda* [70]. The differences in gut microbes between the fifth to sixth instars of *S. frugiperda* may also be related to the development of their immune systems, gut morphology, and gut physicochemical conditions [71,72]. The significant differences in the gut microbes of different developmental stages have also been reported in previous studies on *S. frugiperda* [70]. The dynamic change in gut bacterial communities might help herbivorous insects adapt to the host plants and play an important role in the physiological metabolism of the insects.

To further study the effects of host plants on the diversity of gut bacterial communities, NMDS was used to examine the β diversity. Among samples from four different hosts, all samples of CrB1 were closely clustered together and separately from samples in other treatments, whereas there was an overlap among samples in the other treatments. Therefore, components of CrB1 gut bacterial communities were apparently different from those in larvae fed on other hosts. There were significant differences in the structure of gut bacterial communities in larvae fed on different hosts. The LEfSe analysis effectively detected differentially abundant bacterial taxa in gut microbiomes. A comparison with existing statistical methods and metagenomic analyses of the environmental, gut microbiome, and synthetic data shows that LEfSe analysis consistently provides lower false positive rates and can effectively aid in explaining the biology underlying differences in microbial communities [73]. In general, the results in this study confirm that feeding on different host plants alters the structure of gut bacterial communities in *S. frugiperda* larvae, which is similar to the results for other lepidopterans [56].

PICRUSt2 software was used to analyze the functions of gut bacteria [74]. There were 35 predicted functions of gut bacteria in larvae feeding on different hosts, with most related to metabolic functions. In the analysis of differences in KEGG metabolic pathways, guts of the fifth and sixth instar larvae fed on different hosts were obviously enriched with different functional proteins in most metabolic pathways. In ZmL1 and ZmL2 treatments (larvae fed on corn), samples were enriched with different functional proteins, with genes associated with ABC transport function accounting for the largest proportion. Notably, the bacterial detoxification pump is based on ABC transporters in several main categories: the ABC superfamily [75], the major promoter superfamily [76], and the small multidrug resistance family [77]. The composition of gut bacterial communities in *S. frugiperda* and the ability of members of those communities to metabolize insecticides differ depending on the diversity of chemicals used to treat the host [55]. Accumulation of detoxification and defense genes in the gut of *S. frugiperda* may be related to the diversity of food intake or the variable host environment. The specific factors of influence still need to be verified with further experiments.

This study showed that different host plants had important effects on the structure and diversity of gut bacterial communities in *S. frugiperda*. Host-induced changes in the structure and metabolic functions of gut bacterial communities likely assist *S. frugiperda* larvae in adapting to different food sources. This work provides a good foundation for further exploration of interactions between gut bacteria and hosts for *S. frugiperda*. The results also provide insights into the selection of dominant gut microbial members as potential targets for biological control of the pest. Further research on gut microbes should include more life stages and more host plants, which could provide more perspectives and directions for the adaptive evolution and integrated management of *S. frugiperda*.

## Figures and Tables

**Table 1:** Effective reads data for subsequent analysis after quality control.

Sample Name	Raw Reads *	Clean Reads *	Base (nt)	AvgLen (nt) *	Effective (%) *
HvL1.1	18,434	16,712	24,823,416	1485	90.66
HvL1.2	13,733	12,297	18,259,102	1484	89.54
HvL1.3	12,142	10,472	15,560,783	1485	86.25
HvL1.4	16,263	14,725	21,871,158	1485	90.54
SbL1.1	16,291	14,668	21,773,693	1484	90.04
SbL1.2	20,232	18,538	27,167,447	1465	91.63
SbL1.3	23,156	19,666	29,188,401	1484	84.93
SbL1.4	23,467	21,067	31,264,901	1484	89.77
ZmL1.1	17,639	15,895	23,578,489	1483	90.11
ZmL1.2	10,148	9020	13,191,119	1462	88.88
ZmL1.3	12,649	10,831	15,562,182	1436	85.63
ZmL1.4	13,328	12,007	17,818,003	1483	90.09
CrB1.1	26,445	23,292	34,155,876	1466	88.08
CrB1.2	22,652	20,542	30,485,052	1484	90.69
CrB1.3	18,589	16,033	23,804,488	1484	86.25
CrB1.4	14,176	12,932	18,909,984	1462	91.22
HvL2.1	12,065	11,046	16,295,806	1475	91.55
HvL2.2	15,120	13,924	20,641,745	1482	92.09
HvL2.3	25,269	21,263	31,586,883	1485	84.15
HvL2.4	21,157	19,133	28,414,494	1485	90.43
SbL2.1	15,491	13,452	19,895,164	1478	86.84
SbL2.2	15,980	14,028	20,809,492	1483	87.78
SbL2.3	18,850	16,365	24,214,434	1479	86.82
SbL2.4	25,471	22,426	33,299,817	1484	88.05
ZmL2.1	12,346	11,205	16,617,495	1483	90.76
ZmL2.2	19,422	17,006	25,168,430	1479	87.56
ZmL2.3	17,704	16,464	24,431,320	1483	93
ZmL2.4	13,393	12,090	17,803,176	1472	90.27
CrB2.1	25,248	21,817	32,396,921	1484	86.41
CrB2.2	13,162	11,940	17,728,360	1484	90.72
CrB2.3	16,103	14,615	21,684,846	1483	90.76
CrB2.4	22,175	20,180	29,957,818	1484	91
Total	568,300	505,651	748,360,295	47319	2852.5

* Raw reads represent the number of original reads sequenced by PacBio. Clean reads are the number of high-quality reads obtained after quality control and splicing. AvgLen (nt) is the average sequence length of all samples. Effective (%) is the percentage of effective reads in raw reads.

## Data Availability

The data presented in this study are available in the Supplementary Materials.

## Supplementary Materials

The following supporting information can be downloaded at: https://www.mdpi.com/article/10.3390/genes14020321/s1, Figure S1: Statistics of valid tags and operational taxonomic unit (OTU) clustering of each sample; Table S1: Statistics of valid tags and operational taxonomic unit (OTU) clustering of fifth instar larvae; Table S2: Statistics of valid tags and operational taxonomic unit (OTU) clustering of sixth instar larvae; Table S3: Relative abundance of the most predominant taxa of gut bacteria in different treatment at the phylum level; Table S4: Relative abundance of the most predominant taxa of gut bacteria in different treatment at the genus level; Table S5: α diversity indices of gut bacterial communities in different treatment.
