# Supplementary material for: The Adaptive Evolution in the Fall Armyworm Spodoptera frugiperda (Lepidoptera: Noctuidae) Revealed by the Diversity of Larval Gut Bacteria
Source: Genes (Basel). 2023 Jan 26;14(2):321. doi: 10.3390/genes14020321 (PMC9956290; doi:10.3390/genes14020321)
Supplement: Supplementary file 1 [file genes-14-00321-s001.zip › genes-2068802-supplementary.pdf]

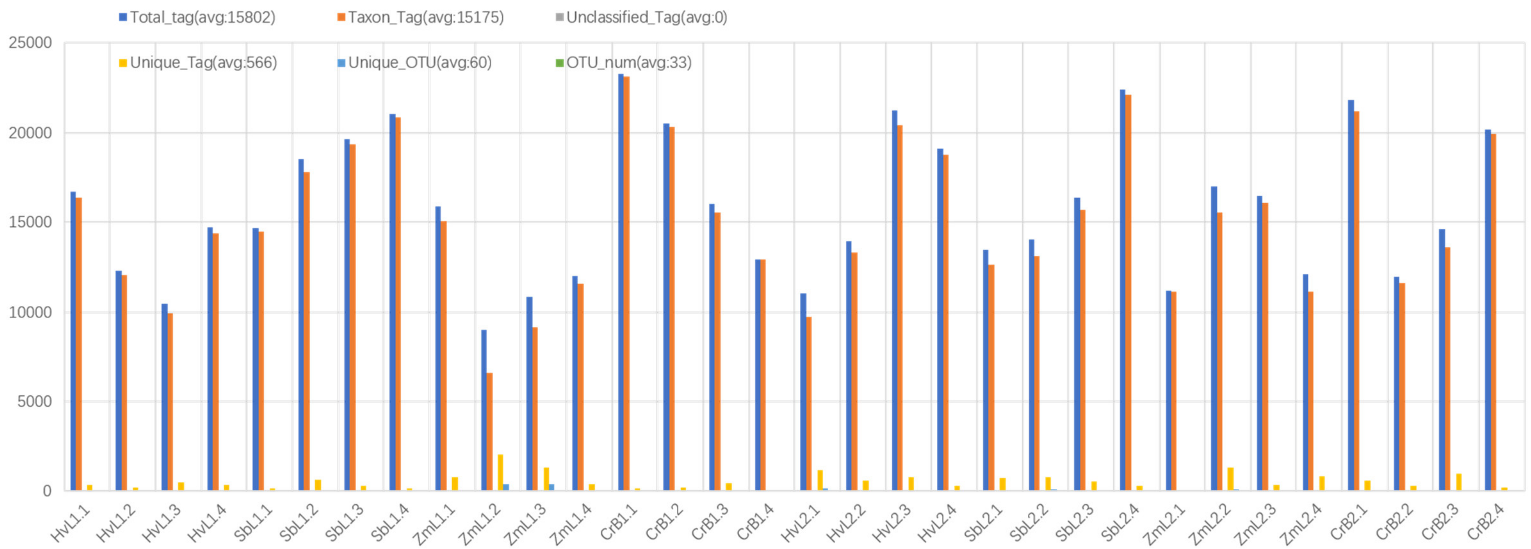

Figure S1. Statistics of valid tags and operational taxonomic unit (OTU) clustering of each sample.

Table S1. Statistics of valid tags and operational taxonomic unit (OTU) clustering of 5th instar larvae.

Sample_Name	Total_Tag	Taxon_Tag	Unclassified_Tag	Unique_Tag	Unique_OTU	OTU_Num
HvL1.1	16712	16341	0	366	5	9
HvL1.2	12297	12046	0	224	27	20
HvL1.3	10472	9942	0	522	8	9
HvL1.4	14725	14372	0	343	10	15
SbL1.1	14668	14490	0	164	14	28
SbL1.2	18538	17827	0	630	81	45
SbL1.3	19666	19343	0	300	23	21
SbL1.4	21067	20865	0	189	13	19
ZmL1.1	15895	15063	0	793	39	48
ZmL1.2	9020	6592	0	2028	400	77
ZmL1.3	10831	9139	0	1306	386	82
ZmL1.4	12007	11554	0	425	28	41
CrB1.1	23292	23128	0	153	11	22
CrB1.2	20542	20320	0	209	13	24
CrB1.3	16033	15528	0	474	31	32
CrB1.4	12932	12924	0	4	4	6
Total	248697	239474	0	8130	1093	498

Table S2. Statistics of valid tags and operational taxonomic unit (OTU) clustering of 6th instar larvae.

Sample_Name	Total_Tag	Taxon_Tag	Unclassified_Tag	Unique_Tag	Unique_OTU	OTU_Num
HvL2.1	11046	9715	0	1178	153	70
HvL2.2	13924	13300	0	594	30	37
HvL2.3	21263	20423	0	818	22	24
HvL2.4	19133	18797	0	329	7	16
SbL2.1	13452	12641	0	768	43	26
SbL2.2	14028	13115	0	792	121	91
SbL2.3	16365	15706	0	575	84	52
SbL2.4	22426	22113	0	290	23	27
ZmL2.1	11205	11154	0	46	5	17
ZmL2.2	17006	15563	0	1306	137	42
ZmL2.3	16464	16087	0	350	27	33
ZmL2.4	12090	11153	0	849	88	37
CrB2.1	21817	21200	0	593	24	25
CrB2.2	11940	11614	0	312	14	21
CrB2.3	14615	13592	0	973	50	31
CrB2.4	20180	19961	0	215	4	13
Total	256954	246134	0	9988	832	562

Table S3. Relative abundance of the most predominant taxa of gut bacteria in different treatment at the phylum level.

Taxonomy	HvL1	SbL1	ZmL1	CrB1	Average	HvL2	SbL2	ZmL2	CrB2	Average	Tax_Detail
Firmicutes	0.9991	0.8462	0.7386	0.5554	0.7848	0.8612	0.8692	0.9376	0.9623	0.9076	k_Bacteria;p_Firmicutes;
Proteobacteria	0.0007	0.1422	0.2274	0.4404	0.2027	0.1187	0.1113	0.0446	0.0284	0.0757	k_Bacteria;p_Proteobacteria;
Bacteroidetes	0	0.0005	0.0028	0.0004	0.0009	0.0183	0.0051	0.0029	0.0003	0.0066	k_Bacteria;p_Bacteroidetes;
Actinobacteria	0.0002	0.0108	0.0232	0.0035	0.0094	0.0018	0.0144	0.0148	0.009	0.01	k_Bacteria;p_Actinobacteria;
Cyanobacteria	0	0	0.0038	0	0.001	0	0	0	0	0	k_Bacteria;p_Cyanobacteria;
Acidobacteria	0	0	0.0007	0	0.0002	0	0	0	0	0	k_Bacteria;p_Acidobacteria;
Verrucomicrobia	0	0.0002	0	0	0.0001	0	0	0	0	0	k_Bacteria;p_Verrucomicrobia;
unidentified_Bacteria	0	0.0001	0.0002	0.0003	0.0001	0.0001	0	0.0002	0	0.0001	k_Bacteria;p_unidentified_Bacteria;
Chloroflexi	0	0	0	0	0	0	0	0	0	0	k_Bacteria;p_Chloroflexi;
Others	0	0	0.0034	0	0.0008	0	0	0	0	0	Others

Table S4. Relative abundance of the most predominant taxa of gut bacteria in different treatment at the genus level.

Taxonomy	HvL1	SbL1	ZmL1	CrB1	Avg.	HvL2	SbL2	ZmL2	CrB2	Avg.	Tax_Detail
Enterococcus	0.9989	0.8460	0.7384	0.5470	0.7826	0.8605	0.8661	0.9329	0.9623	0.9054	k_Bacteria;p_Firmicutes;c_Bacilli;o_Lactobacillales;f_Enterococcaceae;g_Enterococcus;
Ralstonia	0.0005	0.1236	0.0644	0.4333	0.1554	0.0154	0.0006	0.0003	0.0009	0.0043	k_Bacteria;p_Proteobacteria;c_Gammaproteobacteria;o_unidentified_Gammaproteobacteria;f_Burkholderiaceae;g_Ralstonia;
Pseudochrobactrum	0.0000	0.0001	0.0657	0.0000	0.0164	0.0008	0.0001	0.0260	0.0003	0.0068	k_Bacteria;p_Proteobacteria;c_Alphaproteobacteria;o_Rhizobiales;f_Rhizobiaceae;g_Pseudochrobactrum;
Enterobacter	0.0000	0.0003	0.0002	0.0002	0.0002	0.0471	0.0665	0.0001	0.0004	0.0285	k_Bacteria;p_Proteobacteria;c_Gammaproteobacteria;o_Enterobacteriales;f_Enterobacteriaceae;g_Enterobacter;
Klebsiella	0.0000	0.0001	0.0000	0.0000	0.0000	0.0268	0.0378	0.0000	0.0003	0.0162	k_Bacteria;p_Proteobacteria;c_Gammaproteobacteria;o_Enterobacteriales;f_Enterobacteriaceae;g_Klebsiella;
Ochrobactrum	0.0000	0.0151	0.0306	0.0003	0.0115	0.0004	0.0024	0.0015	0.0188	0.0058	k_Bacteria;p_Proteobacteria;c_Alphaproteobacteria;o_Rhizobiales;f_Rhizobiaceae;g_Ochrobactrum;
Alcaligenes	0.0000	0.0002	0.0086	0.0000	0.0022	0.0118	0.0003	0.0032	0.0010	0.0041	k_Bacteria;p_Proteobacteria;c_Gammaproteobacteria;o_unidentified_Gammaproteobacteria;f_Burkholderiaceae;g_Alcaligenes;
Myroides	0.0000	0.0000	0.0018	0.0000	0.0004	0.0102	0.0000	0.0008	0.0001	0.0028	k_Bacteria;p_Bacteroidetes;c_Bacteroidia;o_Flavobacteriales;f_Flavobacteriaceae;g_Myroides;
Achromobacter	0.0000	0.0000	0.0086	0.0000	0.0021	0.0001	0.0001	0.0001	0.0009	0.0003	k_Bacteria;p_Proteobacteria;c_Gammaproteobacteria;o_unidentified_Gammaproteobacteria;f_Burkholderiaceae;g_Achromobacter;

Table S5. Alpha diversity indices of gut bacterial communities in different treatment.

group	observed_species	shannon	simpson	chao1	goods_coverage	PD_whole_tree
CrB1	16	0.882	0.306	21.208	0.999	3.042
CrB2	24	1.351	0.484	31.179	0.999	2.579
HvL1	8	0.899	0.387	11.45	1	1.12
HvL2	27	1.645	0.554	33.156	0.999	2.673
SbL1	24	1.152	0.403	29.936	0.999	2.962
SbL2	40	1.685	0.588	60.073	0.998	4.845
ZmL1	52	2.153	0.654	108.34	0.997	4.724
ZmL2	24	1.14	0.399	27.844	0.999	2.998
